# Reply to Don’t forget to safeguard the lungs during DCD multi-organ retrieval! Early *in situ* oxygenated preservation matters

**DOI:** 10.3389/ti.2026.17755

**Published:** 2026-10-01

**Authors:** Eduardo Miñambres, Dale Gardiner, Helen Opdam

**Affiliations:** 1 Donor Transplant Coordination Unit, Service of Intensive Care, Hospital Universitario Marqués de Valdecilla-IDIVAL, School of Medicine, Universidad de Cantabria, Santander, Spain; 2 Organ and Tissue Donation and Transplantation, NHS Blood and Transplant, Bristol, United Kingdom; 3 National Medical Director, Organ and Tissue Authority, Canberra & Intensive Care Specialist, Austin Health, Melbourne, Australia

**Keywords:** DCDD (donation after circulatory determination of death), dead donor rule, ESOT, lung transplant, ventilatory management

We read with interest the letter submitted by Jorda-Aragón et al [1] and appreciate their thoughtful comments. However, we do not agree with the authors’ assertion that the lung has been relegated to a secondary role in the Bucharest consensus documents [2, 3], which established standards for controlled donation after circulatory determination of death (cDCDD) in adults and provided specific recommendations regarding the use of normothermic regional perfusion (NRP) in this donor population. These consensus processes were not intended to provide detailed, organ-specific technical guidance, except where such guidance was directly relevant to the application of NRP. The management of lungs for transplantation in the context of both abdominal NRP (A-NRP) and thoracoabdominal NRP (TA-NRP) is appropriately addressed in the NRP consensus document.

The authors’ suggest that early lung ventilation, immediately after completion of the mandatory hands-off period and declaration of death, is essential to optimize lung graft preservation [1]. We fully support measures aimed at achieving early optimal lung preservation. Indeed, there is international experience with combined lung recovery and A-NRP having been reported, including published protocols in which mechanical ventilation is resumed soon after death has been formally declared in combination with initiation of A-NRP [4–6]. Conversely, concerns exist in some jurisdictions that the immediate resumption of cyclical ventilation could potentially be associated with restoration of effective cardiac activity and safety measures are required to delay the resumption of mechanical ventilation beyond the completion of the hands-off period. These protocols allow for immediate reintubation but require delay of lung insufflation until 10 min after the onset of mechanical asystole and cyclical ventilation only during the pulmonary flush at surgical retrieval [7, 8].

In the context of A-NRP, monitoring of the left radial artery during cDCDD is used as a simple means of detecting any restoration of effective cardiac activity and systemic circulation or the possibility of restoring cerebral perfusion due to inadequate occlusion of the thoracic aorta. Such an event would be readily identified by the appearance of a pulsatile arterial pressure waveform in the left radial artery in the event of restoration of effective cardiac activity, or by the appearance of non-pulsatile pressure in the left radial artery in the event of inadequate occlusion of the thoracic aorta [9, 10].

These differences highlight the importance of ongoing evaluation of post-mortem interventions and the publication of case series or incident sharing. As international clinical experience continues to accumulate, greater harmonization of practices across countries may become possible. However, the specific timing of the resumption of mechanical ventilation following the determination of death was not addressed during the Bucharest consensus deliberations and, consequently, no specific recommendation on this issue was made.

We are aware of the lack of standardization in the combined recovery of the heart and lungs using TA-NRP. As the authors point out, the ESOT Bucharest Consensus explicitly acknowledges the potential risks of lung congestion and cardiac distension during TA-NRP. Left atrial venting, through the insertion of a venting catheter via the interatrial groove at the initiation of TA-NRP, has been proposed to prevent hydrostatic pulmonary edema [11]. However, although this issue was discussed during the Consensus Conference, no consensus was reached among the panelists. Therefore, while acknowledging this concern, we cannot recommend this maneuver based on the findings of the Delphi process. As international experience with the simultaneous recovery of the heart and lungs using TA-NRP continues to grow, the technique may become increasingly standardized, allowing optimal preservation of all organs.

We completely agree with the authors that the lung should not be regarded as the “forgotten organ” in regional perfusion strategies or cDCDD consensus guidelines. Protocols should incorporate measures to optimize lung oxygenation and preservation while taking advantage of the lung’s distinctive physiological characteristics. At the same time, the acceptability and implementation of specific interventions may therefore remain context-dependent and influenced by the regulatory framework.

## Data Availability

The original contributions presented in the study are included in the article/supplementary material, further inquiries can be directed to the corresponding author.
